# Cloud-Based Personalized sEMG Classification Using Lightweight CNNs for Long-Term Haptic Communication in Deaf-Blind Individuals

**DOI:** 10.3390/bioengineering12111167

**Published:** 2025-10-27

**Authors:** Kaavya Tatavarty, Maxwell Johnson, Boris Rubinsky

**Affiliations:** Department of Mechanical Engineering, University of California, Berkeley, CA 94720, USA

**Keywords:** Usher syndrome, surface electromyography, arm sleeve, communication

## Abstract

Deaf-blindness, particularly in progressive conditions such as Usher syndrome, presents profound challenges to communication, independence, and access to information. Existing tactile communication technologies for individuals with Usher syndrome are often limited by the need for close physical proximity to trained interpreters, typically requiring hand-to-hand contact. In this study, we introduce a novel, cloud-based, AI-assisted gesture recognition and haptic communication system designed for long-term use by individuals with Usher syndrome, whose auditory and visual abilities deteriorate with age. Central to our approach is a wearable haptic interface that relocates tactile input and output from the hands to an arm-mounted sleeve, thereby preserving manual dexterity and enabling continuous, bidirectional tactile interaction. The system uses surface electromyography (sEMG) to capture user-specific muscle activations in the hand and forearm and employs lightweight, personalized convolutional neural networks (CNNs), hosted on a centralized server, to perform real-time gesture classification. A key innovation of the system is its ability to adapt over time to each user’s evolving physiological condition, including the progressive loss of vision and hearing. Experimental validation using a public dataset, along with real-time testing involving seven participants, demonstrates that personalized models consistently outperform cross-user models in terms of accuracy, adaptability, and usability. This platform offers a scalable, longitudinally adaptable solution for non-visual communication and holds significant promise for advancing assistive technologies for the deaf-blind community.

## 1. Introduction

Deaf-blindness is a unique and profoundly disabling condition characterized by the combined loss of hearing and vision, which severely limits communication, access to information, orientation and mobility, and daily independence. The spectrum of deaf-blindness is broad, encompassing individuals who are born with the condition as well as those who acquire it later in life due to genetic disorders, infections, trauma, or age-related decline. Although precise global estimates are difficult to determine due to inconsistent definitions and reporting standards, it is estimated that between 0.2% and 2% of the world’s population is deaf-blind [1], with over two million adults in the United States alone reporting combined severe hearing and vision loss [2,3]. The prevalence increases sharply with age; approximately 8.6% of adults over the age of 70 report difficulties with both hearing and vision [4,5].

**Figure 1:**
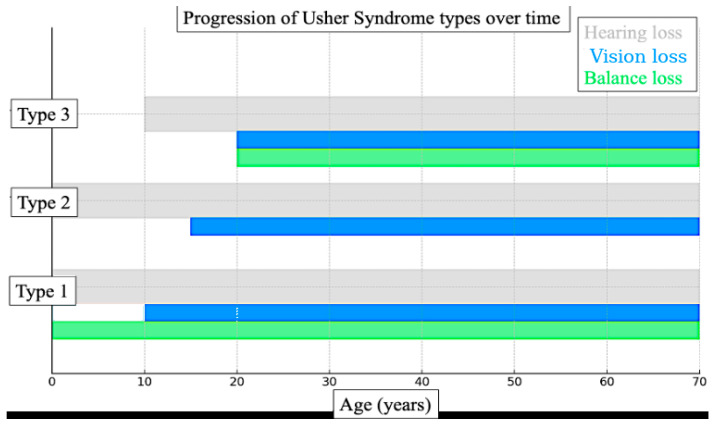
Diagram illustrating the typical progression of Usher syndrome by type. It shows the onset and duration of hearing loss (grey), vision loss (blue), and balance issues (green) across the lifespan for Types 1, 2, and 3.

The etiologies of deaf-blindness are diverse. Usher syndrome, the most common genetic cause, results in sensorineural hearing loss combined with progressive vision loss due to retinitis pigmentosa. Certain subtypes also involve balance impairments [6,7]. Figure 1 illustrates the typical progression of Usher syndrome by type, showing the onset and duration of hearing loss, vision loss, and balance difficulties across the lifespan for Types 1, 2, and 3. This diagram is particularly relevant to the technology developed in this study, as it highlights the need for adaptable communication solutions that evolve alongside the sensory changes experienced by individuals with Usher syndrome.

Other genetic syndromes that lead to combined vision and hearing loss include CHARGE, Alström, Wolfram, and Norrie diseases, each contributing to dual sensory impairment through distinct molecular mechanisms [8,9,10]. Infections such as congenital rubella and cytomegalovirus (CMV) also remain significant causes, particularly in regions lacking widespread maternal vaccination or comprehensive prenatal screening programs [11,12]. Additional contributors include traumatic brain injury, peroxisomal and mitochondrial disorders, and, increasingly, age-related conditions such as presbycusis and macular degeneration [13,14,15,16,17].

**Figure 2:**
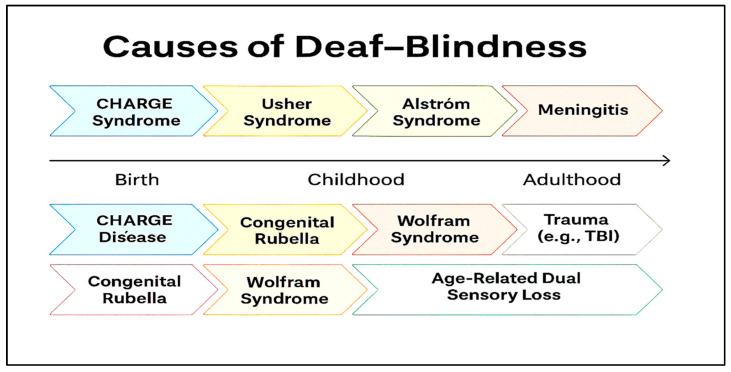
Various etiologies of deaf-blindness and the stage in life when they may appear.

Figure 2 presents a synthesized overview of the various causes of deaf-blindness, mapped against the typical stage of life at which they manifest. While the technology developed in this study may ultimately support individuals affected by all these etiologies, we believe its most immediate applications lie in addressing the needs of those with Usher syndrome and those experiencing age-related dual sensory loss.

Regardless of etiology, individuals who are deaf-blind face formidable challenges across nearly every domain of life. Among these, communication remains one of the most significant and persistent barriers, given the dual absence of or reduction in both auditory and visual channels. Many deaf-blind individuals cannot access spoken or visual languages and instead rely on tactile-based methods tailored to their remaining sensory abilities. Traditional spoken language and visual sign language are often inaccessible, necessitating the development and adoption of alternative communication systems rooted in touch.

Tactile communication methods form the foundation of interaction for many deaf-blind individuals. One of the most widely used approaches is tactile sign language, such as hands-on American Sign Language (ASL), in which standard signs are received through touch by placing one’s hands over those of the signer to perceive movement, shape, and position [18]. This method is often adapted to accommodate a user’s residual sensory capabilities and tactile preferences. Other direct tactile methods include the Tadoma method, where one places their fingers on a speaker’s lips, jaw, and throat to feel articulatory movements, and the print-on-palm technique, in which letters are traced onto the palm with a finger. Print-on-palm is especially effective in one-on-one interactions, early education, or when linguistic exposure is limited [19]. Symbolic and text-based systems also play a critical role. Braille, a widely adopted system of raised dots, remains a cornerstone of literacy and independent communication. Modern tools like refreshable Braille displays and Braille notetakers have significantly expanded access to digital content [20,21,22]. For users with cognitive disabilities or minimal formal language acquisition, tactile symbols, such as miniature objects or parts of objects, can represent people, places, or actions, offering a concrete and intuitive means of communication. Augmentative and Alternative Communication (AAC) systems have also been adapted for tactile use. These include tactile communication boards, speech-generating devices with touch-based interfaces, and apps that convert text to Braille or provide haptic feedback. Some advanced AAC platforms incorporate object-based symbols and tactile iconography to support individuals with varying cognitive and developmental profiles [23].

Collectively, these communication strategies form a spectrum of tactile approaches tailored to the unique needs of each individual. While no single method is universally applicable, each offers a vital bridge to interaction, education, and autonomy when appropriately matched to a user’s abilities and preferences. Yet despite these options, access to information remains limited. Digital media often lacks compatibility with Braille displays or screen readers, and educational or employment opportunities are frequently hindered by the need for individualized accommodations and the availability of trained support personnel such as interveners or support service providers (SSPs). These structural barriers are compounded by social isolation, mental health challenges, and inadequate access to healthcare systems that are not equipped for dual sensory loss [24,25].

Recent advances in assistive technologies offer promising tools to bridge these gaps. Devices such as Braille notetakers, vibration-based alert systems, and GPS units with haptic interfaces are increasingly used to enhance independence and environmental awareness. Smartwatches like the Dot Watch [26] provide real-time tactile feedback for notifications and timekeeping. However, these devices often fall short in delivering fluid, bidirectional, and context-aware communication, particularly in dynamic environments or for users relying on tactile signing.

Emerging technologies are beginning to address these limitations. Artificial intelligence (AI)-enabled gloves embedded with sensors have demonstrated potential for recognizing tactile sign language and translating gestures into speech or text in real time [27]. Haptic augmented and virtual reality (AR/VR) platforms provide immersive educational experiences, simulating 3D tactile environments, Braille, and mid-air haptic feedback using ultrasonic arrays [28,29]. Novel interfaces such as TanvasTouch [30], which uses surface-based electrostatic friction to simulate texture on flat screens, and Ultraleap’s mid-air haptics [31], which allows users to perceive tactile shapes and directional cues without contact, are especially promising for STEM education and digital exploration, fields in which deaf-blind individuals have been historically underrepresented.

Nonetheless, many of these sensing technologies are slow, require physical proximity, and are not yet optimized for real-world usability [32]. Most AI models for sign language recognition are trained on visual ASL datasets, not tactile variants, and haptic systems often lack the resolution, bidirectional responsiveness, or ergonomic design needed for extended use. Challenges such as glove comfort, battery life, and the scarcity of tactile-specific datasets further limit widespread deployment. Overcoming these barriers demands user-centered design, robust usability testing, and sustained collaboration with deaf-blind individuals to ensure that technologies meet their needs in authentic contexts.

Despite decades of progress, the foundational insight expressed by Kramer and Rosenfeld in 1975 remains strikingly relevant today: “We think that the most critical factor that we may have to convey to you is that no matter how advanced a person’s communication skills are, it is impossible for a deaf-blind person to communicate with another human being without that other person using a special communication skill. All the communication skills in the world cannot help if there isn’t another person to talk to. At this time, the world of the deaf-blind person is limited to the length of his arms. This is a critical factor which is very difficult for most of us to comprehend. Once there was a story of a deaf-blind man who was asked what his wish would be if he had only one to make, and he said, ‘I wish for arms a mile long.’” [33]. This profound statement underscores the ongoing need not only for technological advancement but also for societal commitment to inclusive communication—anchored in empathy, collaboration, and mutual skill.

**Figure 3:**
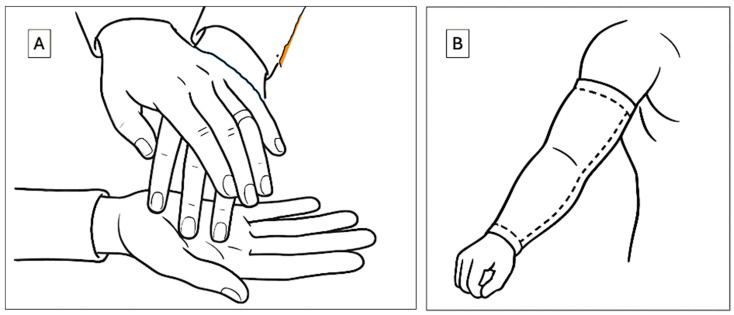
Haptic modes of communication. (**A**) Current method that employs hand touch (**B**) the method of this study that uses an arm sleeve for communication.

The overarching goal of our research is to develop technologies that enable individuals who are deaf-blind to communicate and interact more effectively with the broader world. Figure 3A illustrates a representative example of current sensory communication practices used in interactions with deaf-blind individuals. These methods typically rely on direct physical contact, with the hands serving as both the receptive and expressive medium for tactile communication.

The hands are frequently chosen as the primary site for this interaction due to their dense sensory innervation, which allows for precise tactile perception. However, this mode of communication presents two major limitations. First, it necessitates close physical proximity between communication partners, thereby limiting its use in dynamic, public, or remote settings. Second, it occupies the hands, an anatomical structure essential for a wide range of daily functional tasks, thereby restricting the user’s independence and mobility during communication.

At its core, successful communication between individuals depends on an effective interface between a sensory input site and a sensory output site. While current tactile-based systems fulfill this role within limited contexts, they remain constrained by anatomical dependency and spatial proximity. To overcome these challenges, our research focuses on the development of alternative, wearable haptic technologies that decouple the communication interface from the hands. By relocating this interface to other areas of the body, such as the arms, we aim to enhance user autonomy, preserve manual dexterity, and expand the spatial and functional flexibility of communication for individuals who are deaf-blind.

The technology described herein introduces several novel haptic innovations. Most notably, we propose the use of the arm, augmented with a wearable sleeve, as an alternative site for sensory communication (Figure 3B). This approach offers multiple advantages over traditional hand-based systems. The arm provides a significantly larger surface area than the hand, creating an expanded interface for the delivery and reception of haptic stimuli. Crucially, the use of an arm sleeve preserves hand functionality, allowing users to perform other tasks concurrently without interference. The sleeve is non-obstructive, compatible with a wide variety of clothing, and has already been explored in therapeutic applications, including interventions for individuals with autism [34]. Although the tactile spatial resolution of the arm is inherently lower than that of the hand, due to its reduced density of mechanoreceptors, the increased surface area enables the distribution of signals over a broader canvas. This spatial advantage supports the encoding of complex haptic patterns in both space and time. Recent advances in actuator design and signal encoding techniques can further compensate for the arm’s lower native acuity, enabling reliable discrimination of stimuli when user training and adaptive interface strategies are employed. Thus, while the arm has lower intrinsic tactile resolution, its effective resolution for haptic communication can be significantly enhanced through engineering design and user adaptation.

We have previously introduced the use of arm-worn bracelets as a platform for bidirectional communication [35,36]. The technologies described in those studies employed haptic sensing on the arm for message reception and kinematic movements of the arm for message transmission. In that implementation, localized vibration served as the primary haptic excitation modality, while arm rotation was used to convey kinematic signals. Testing with student participants demonstrated that the bracelets could be comfortably worn for extended periods without interfering with daily activities.

Building upon these findings, we propose that the arm remains a viable and effective site for both receiving and transmitting haptic communication. In the present study, we aim to expand the range of expressive modalities beyond kinematic motion by integrating electrophysiological signals, specifically surface electromyography (sEMG) recorded from the arm, as an additional communication channel. By incorporating sEMG as an expressive modality, we seek to significantly broaden the set of communicable elements and establish the feasibility of using physiological signals as a novel, user-driven input dimension in haptic communication systems designed for individuals who are deaf-blind.

Surface electromyography (sEMG) is a noninvasive technique used to measure the electrical activity generated by skeletal muscles. By placing electrodes on the skin’s surface directly above the muscle(s) of interest, sEMG sensors detect voltage differences produced by the depolarization and repolarization of muscle fibers during contraction and relaxation [37]. This technique enables the simultaneous monitoring of multiple muscles, making it a powerful tool for analyzing patterns of muscle activation during coordinated movements [38]. By cataloging these activation patterns, researchers can accurately infer the relationship between upstream neural signals and downstream motor outputs in the form of specific gestures or actions.

In recent years, the integration of deep learning techniques has significantly enhanced the utility of sEMG by enabling automated interpretation of large, high-dimensional datasets. These systems are now commonly used to classify complex gestures with increasing accuracy [39]. sEMG-based gesture recognition has gained traction in assistive technologies and medical robotics, particularly in applications such as neuroprostheses, where it has been shown to improve the control, fluidity, and functionality of arm and leg prosthetic devices [40,41]. Traditional nonverbal communication methods, such as sign language, depend on fine motor control and precise coordination of hand and arm muscles to form gestures that represent letters, numbers, words, or phrases. When performed sequentially, these gestures enable real-time communication for individuals with hearing impairments. By leveraging sEMG to monitor the patterns of muscle excitation underlying these gestures, it becomes feasible to develop systems that interpret and translate muscle activity directly into meaningful linguistic outputs [42].

Figure 1 and Figure 2 illustrate that in many cases, particularly among individuals with Usher syndrome and those with age-related dual sensory impairment, both vision and hearing loss are progressive. This is especially pronounced in children diagnosed with Usher syndrome, for whom early intervention is essential.

Functional magnetic resonance imaging (fMRI) studies have compared neural activation patterns associated with tactile word recognition across three modalities: Braille, Print-on-Palm (PoP), and a haptic variant of American Sign Language (haptic ASL or hASL). These studies reveal that all three tactile communication modes elicit robust activation in the occipital cortex and in classical language-processing regions, including the posterior superior temporal and inferior frontal areas of the left hemisphere [43]. These findings highlight the neurological plasticity that supports tactile language acquisition and suggest the value of introducing personalized haptic communication systems early in individuals with progressive dual sensory loss.

While most current research in sEMG-based gesture recognition focuses on generalized AI systems designed for broad applicability, we propose a personalized, adaptive approach tailored specifically to individuals with Usher syndrome and age-related sensory decline. Our method involves a long-term sEMG-based gesture classification system that can accommodate physiological changes over time. This system employs lightweight, user-specific machine learning models capable of adapting to individual variations in muscle activation, both across users and throughout a single user’s life.

The primary focus of this paper is to introduce the technological foundations of this personalized system and present preliminary findings from a pilot study involving a small cohort of participants. These early results demonstrate the feasibility and potential of this approach for enabling fluid, scalable, and longitudinally adaptable haptic communication for individuals with combined visual and auditory impairments.

## 2. Materials and Methods

**Figure 4:**
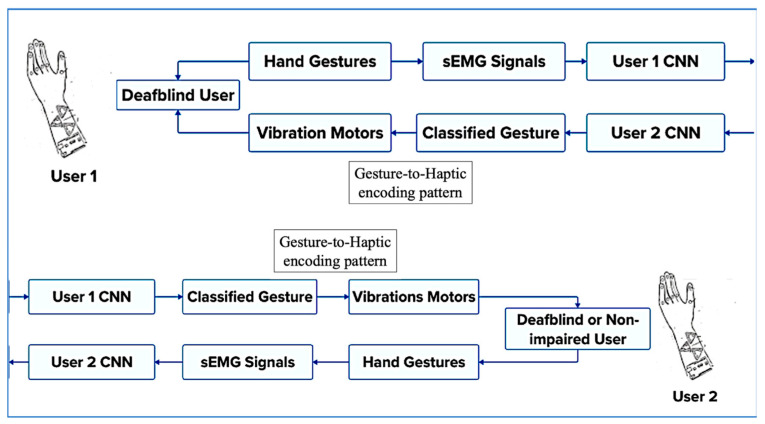
Overall communication architecture between two users of the device.

The overall communication architecture between two users of the technology introduced in this study is illustrated in Figure 4. As shown, the system enables communication between two individuals who are deaf-blind, or between a deaf-blind user and a non-impaired communication partner. The process begins when the first user performs a tactile or visual sign language gesture, such as a TSL or an ASL sign. The resulting muscle activations are recorded by surface electromyography (sEMG) sensors and classified to identify the performed gesture. This gesture is then mapped to a specific haptic feedback pattern, which is delivered to the second user through vibrotactile stimulation on the fingertips, enabling real-time, bidirectional communication.

A standard sEMG setup consists of three core components: surface electrodes, a signal processing unit, and a software interface for data interpretation. Electrodes are positioned on the skin above key forearm muscles involved in hand and arm gestures, such as the flexor and extensor muscle groups.

The signal processing unit amplifies the low-amplitude electrical signals generated by muscle fibers and filters out ambient noise to preserve signal integrity. These filtered signals are subsequently processed by the software interface, which employs real-time classification algorithms to interpret the user’s muscle activation patterns. Each recognized gesture is mapped to a unique sequence of vibration patterns, similar to the communication modality described in [35], and transmitted to the wearable interface of the deaf-blind recipient, thereby effectively conveying the intended message. These mappings between gestures and vibration sequences are fully customizable, enabling support for various sign languages, dialects, or user-specific communication schemes. In scenarios involving communication between a deaf-blind individual and a sighted or hearing person, a computer can serve as the receiving and transmitting interface of the sighted or hearing person user’s end, allowing seamless bidirectional communication between users with different sensory profiles.

Surface electromyography enables detailed analysis of muscle excitation patterns, which can be used to classify discrete hand gestures. However, a known limitation of sEMG-based systems is the high degree of inter-user variability in signal patterns, which has posed challenges for the development of generalizable deep learning models. In many applications, this variability hinders the performance of “one-size-fits-all” classifiers.

In the context of our application, designed to support a single user over the course of their lifetime, physiological variability becomes an advantage rather than a limitation. Because the system is personalized and continuously adapted to the same individual, it can accommodate the progressive changes associated with aging and sensory decline, such as muscle atrophy, strength variation, and shifts in motor control. This is particularly important for individuals who experience gradual vision loss over time, as in the case of Usher syndrome.

To enable this long-term adaptability, we have developed a centralized, cloud-based sEMG hand gesture classification framework that employs lightweight, user-specific convolutional neural networks (CNNs). These personalized models are designed to accommodate evolving signal patterns and can be efficiently updated with minimal retraining, ensuring sustained performance as the user’s muscle physiology changes throughout their lifespan.

The proposed system employs machine learning techniques to classify hand gestures using multi-channel surface electromyography (sEMG) data and is designed to meet several key criteria critical for practical deployment and long-term usability. First, the system supports real-time performance, enabling low-latency classification of fluid, continuous hand movements to ensure seamless and intuitive user interaction. Second, it incorporates user adaptability, allowing the system to accommodate a wide range of users with varying muscle geometries and remain robust to minor inconsistencies in electrode placement.

Third, the system offers gesture flexibility, with the ability to adapt to evolving gesture sets. New gestures can be incorporated with minimal training data and short retraining times, allowing the system to evolve alongside the user’s communication needs. Fourth, the architecture emphasizes portability and accessibility through a centralized server model that facilitates storage, retrieval, and deployment of personalized user profiles across different platforms and devices. Finally, the system supports personalized adaptation through AI-driven updates that adjust to changes in an individual’s physiology over time, ensuring sustained performance and long-term relevance.

### 2.1. Hardware and System Design

To illustrate the underlying concepts and demonstrate feasibility, we designed, built, and tested a prototype system consisting of four surface electromyography (sEMG) sensors that record muscle activation signals. These sensors are connected to an Arduino R4 Wi-Fi microcontroller, which transmits the data to a centralized server for both model training and real-time gesture recognition.

### 2.2. Hardware Setup

We utilize the MyoWare 2.0 Ecosystem as the primary platform for acquiring surface electromyography (sEMG) signals [44]. While the system is adaptable to varying numbers of electrodes, we selected four muscle sensors for this prototype as an illustrative example. Each MyoWare muscle sensor has an input impedance of 800 ohms, operates with a typical supply voltage of 3.3–5.0 V, and features a high common-mode rejection ratio (CMRR) of 140 dB, ensuring accurate signal acquisition in noisy environments.

The gain for each sensor, in the envelope detection mode, is adjustable using an onboard potentiometer according to the relationship:

G = 200 × R/1 kΩ

where *R* is the resistance (kΩ) set by the potentiometer.

To minimize noise and preserve the fidelity of the sEMG signals, the system incorporates a series of filters. An active high-pass filter (1st order, fc = 20.8 Hz, –20 dB/decade) eliminates low-frequency drift and motion artifacts, while an active low-pass filter (1st order, fc = 498.4 Hz, –20 dB/decade) removes high-frequency noise. Given that sEMG signals typically fall within the range of 0–10 mV and 10–500 Hz, this 20–500 Hz passband effectively retains the relevant signal content while suppressing unwanted noise and motion-related interference [44,45,46].

**Figure 5:**
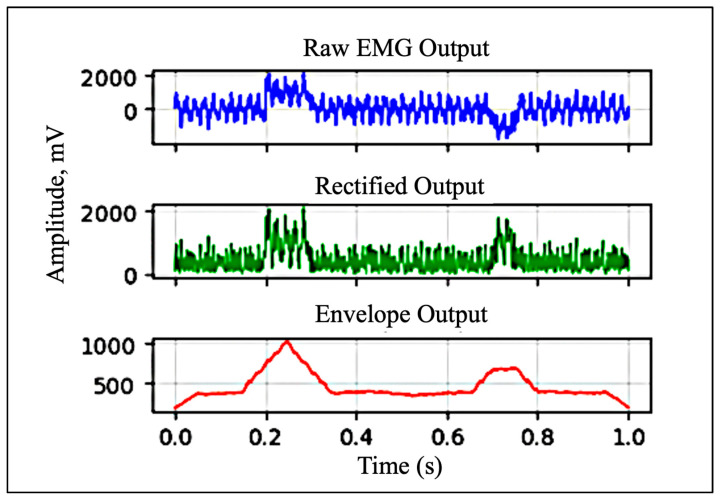
Raw, rectified, and envelope signals (data shown is simulated).

For envelope detection, the system uses a passive low-pass filter with a cutoff frequency of 3.6 Hz to smooth the rectified signal, capturing the overall shape and amplitude modulation of muscle activity. MyoWare sensors can output data in three formats: raw (RAW), rectified (RECT), or envelope (ENV). Since the envelope signal is the primary output of the sensor and is most suitable for gesture classification tasks, the ENV output was used in this study. Figure 5 illustrates the differences among these output formats and the role each plays in interpreting muscle activity.

The MyoWare muscle sensors acquire analog sEMG signals, which are read by the Arduino R4 Wi-Fi via its onboard analog-to-digital converter (ADC). Thanks to MyoWare’s integrated multi-stage filtering architecture, the acquired signals require no additional denoising or post-processing prior to analysis. Each muscle sensor employs a bipolar electrode configuration, with two input electrodes placed over the target muscle and one reference (ground) electrode positioned on a nonadjacent muscle group or bony surface. This arrangement improves signal fidelity by enhancing differential amplification and reducing ambient electrical noise.

In this setup, four MyoWare muscle sensors are connected to the Arduino R4 Wi-Fi through link shields, which attach to the sensors via snap connector mechanisms. TRS (Tip-Ring-Sleeve) cables are used to interface each link shield with an Arduino shield that routes the signals to the appropriate analog input pins on the microcontroller. For optimal signal quality, single-use wet electrodes were employed in this study. However, alternative electrode options, including reusable dry electrodes and conductive fabric sleeves, could be considered in future implementations to enhance user comfort and long-term wearability.

The sensors are powered with a 5V supply from a dedicated battery pack. This battery-based power source was chosen to minimize electrical noise, which was observed when the sensors were powered via USB from a personal computer or from AC mains. To further reduce potential interference and signal saturation, the use of a USB isolator with the MyoWare 2.0 Ecosystem may warrant investigation in future studies.

### 2.3. Electrode Placement

Accurate electrode placement is a critical factor in obtaining reliable surface electromyography (sEMG) data [47]. Electrodes must be positioned over muscles that are directly involved in performing the intended coordinated movements to ensure that recorded signals reflect meaningful neuromuscular activity.

A common challenge in sEMG acquisition is crosstalk, which refers to interference caused by electrical activity from adjacent or underlying muscles. Crosstalk can compromise signal specificity and reduce the accuracy of gesture classification. Its impact can be minimized through thoughtful electrode placement and effective signal filtering. Specifically, wider spacing between electrodes decreases muscle selectivity, whereas closer spacing enhances the precision of muscle-specific signal capture [47].

The human forearm and wrist contain over 20 muscles, many of which contribute to the fine motor control of the fingers [48]. These include both superficial muscles, such as the flexor digitorum superficialis (FDS), and deeper muscles, such as the abductor pollicis longus (APL). Due to the limitations of surface electrodes in capturing signals from deeper tissues, our system focuses primarily on superficial muscles to maximize signal quality and interpretability.

In this study, we target three forearm muscles that are highly relevant to finger and thumb gestures: (a) Flexor Digitorum Superficialis (FDS): responsible for flexing the middle phalanges of digits 2–5 at the proximal interphalangeal (PIP) joints, and assisting in flexion at the metacarpophalangeal (MCP) joints, (b) Extensor Digitorum (ED): facilitates extension of the four fingers at the MCP joints, (c) Flexor Pollicis Longus (FPL): controls thumb flexion at both the interphalangeal (IP) and MCP joints, enabling the curling motion of the thumb.

Although the FPL is located in a deeper compartment of the forearm, we placed an electrode over its anatomical location and observed clear signal spikes during thumb flexion. Through empirical testing, we found that the combination of FDS, ED, and FPL provides sufficient discriminatory power for recognizing a range of common fine motor gestures.

**Figure 6:**
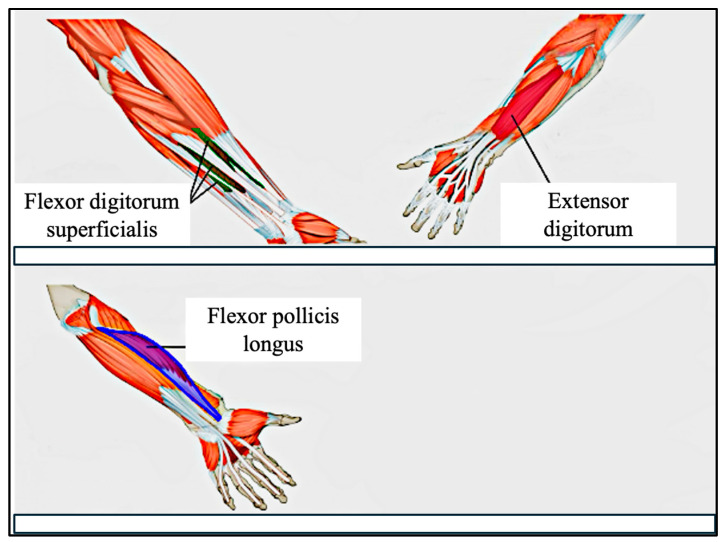
Electrode Placement on the forearm. Top image shows superficial muscles, and lower image shows deep muscles.

Figure 6 illustrates the electrode placement strategy used in our study, highlighting the anatomical locations of these three targeted muscles.

### 2.4. Data Processing

Sensor data is standardized using the StandardScaler function from the scikit-learn library [49]. This function normalizes the sEMG signals by subtracting the mean and scaling to unit variance, ensuring that the transformed data has a mean of 0 and a standard deviation of 1.

Standardization improves model convergence and stability, particularly when working with neural networks and gradient-based learning algorithms.

Each data point is transformed according to the equation below, where x is the original signal value, μ is the mean, σ is the standard deviation, and z is the standardized value:

z = (x − μ)/σ(1)

The mean (μ) and standard deviation (σ) are calculated during the training phase and stored. These same values are then reused during model deployment to ensure consistent scaling across both training and inference, thereby maintaining compatibility between incoming data and the trained model’s expectations.

### 2.5. Signal Preprocessing Timeline

(1) Window size: 32 samples with 50% (2) Normalization: Z-score standardization (3) Filtering: 20–500 Hz bandpass filter (4) Channels: All 4 channels retained without fusion or discard (5) Sampling rate: 50 Hz

### 2.6. Model Architecture

**Figure 7:**
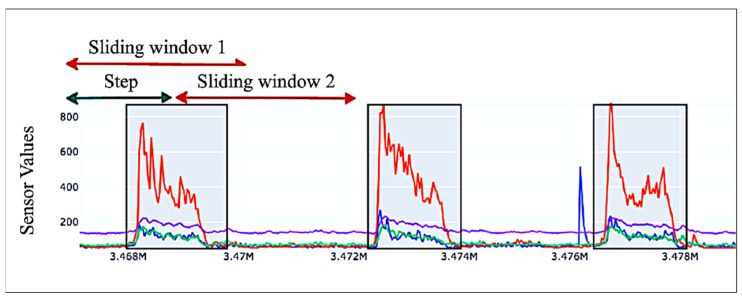
Sliding window mechanism shown on sample sEMG data from 4 sEMG sensors. Each color represents a sensor channel.

To enable real-time gesture classification, we developed a one-dimensional Convolutional Neural Network (1D-CNN) that processes normalized sEMG sensor data along with temporal context derived from preceding sensor readings. The temporal context is captured using a sliding window approach, in which the model considers the past *N* = 32 data points for each prediction. This technique allows the model to learn spatiotemporal patterns, which are essential for recognizing fluid and continuous gestures, such as those found in sign language. An illustration of the sliding window technique is provided in Figure 7.

This temporal framing reduces the need for extensive hand-crafted feature engineering, as the CNN learns the necessary representations directly through learned channel filters.

The architecture consists of two sequential 1D convolutional layers (Conv1D), each followed by ReLU activations and max-pooling operations, which extract and condense temporal features from the input sequence. These convolutional layers are followed by a stack of five fully connected (dense) layers, which progressively reduce the feature space and map it to the final classification output. The number of neurons in the final layer is dynamically determined based on the number of gesture classes defined for each user.

**Figure 8:**
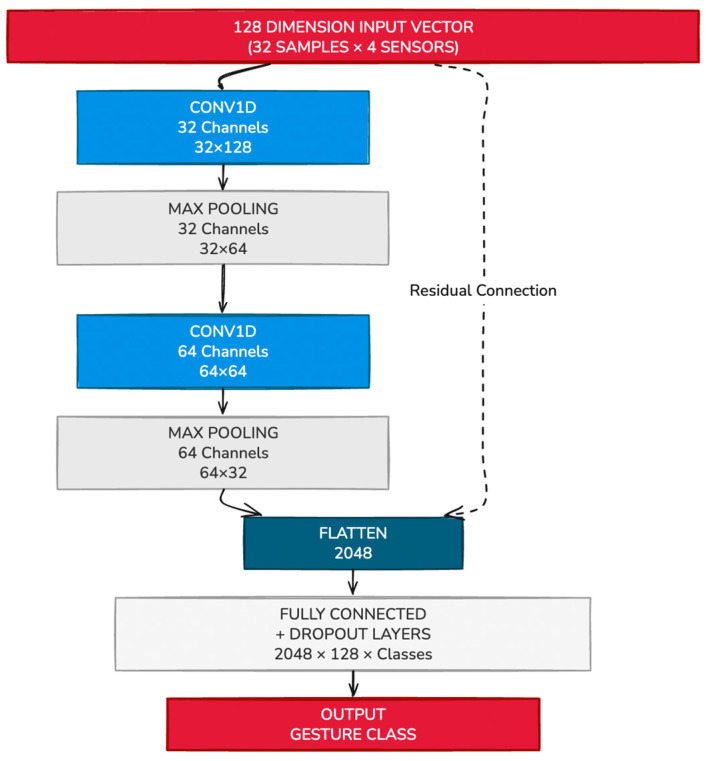
Example CNN Network to classify sEMG signals from 4 sensors into gesture classes.

The network architecture is implemented in PyTorch version 2.3.1, with design flexibility that allows personalization based on the number of sensors used and the size of the gesture set. The complete CNN architecture is depicted in Figure 8.

### 2.7. Training Procedure

The model is trained using the Adam optimizer with a learning rate of 0.001, which provides efficient and adaptive gradient updates for deep learning models. The CrossEntropyLoss function is used as the loss criterion, appropriate for multi-class classification tasks involving mutually exclusive gesture classes.

Training is conducted in mini batches of size 32, with gradient updates performed after each batch. The model is compiled and optimized for performance, leveraging hardware acceleration where available. The training loop runs for a minimum of 10 epochs, or until early stopping is triggered based on the absence of improvement in validation loss. To ensure effective performance monitoring, loss and accuracy metrics are computed at regular intervals during training. This enables real-time evaluation of the model’s learning behavior and generalization to unseen data.

### 2.8. System Architecture and Personalization

#### User Personalization

The inherent variability of surface electromyography (sEMG) signals across individuals presents a major challenge for generalized machine learning models. While some cross-user models may perform adequately among users with similar physiological characteristics, they typically lack the flexibility needed to accommodate a diverse user base. In applications such as prosthesis control and personalized communication systems, where users exhibit substantial differences in muscle geometry, anatomy, and motor patterns, it is essential to implement systems that can be tailored to both the physiological traits and functional needs of each individual—using minimal training data.

This study presents a novel approach to personalization through the use of highly customizable, small-scale convolutional neural networks (CNNs) in conjunction with a centralized server-based architecture. This framework supports long-term adaptability by allowing individual models to evolve in response to physiological changes over a user’s lifetime.

Due to the compact nature of the proposed CNN architecture, model storage and retrieval pose no significant computational burden. Each user generates a personalized model, which enables the definition of custom gesture sets, including gestures not previously encountered by the system. The system is designed to be robust to moderate variations in electrode placement and anatomical differences, challenges that often degrade the performance of standard cross-user or even static single-user systems. By contrast, our system adapts effectively across a broad spectrum of users and use cases.

In addition, users can dynamically edit their gesture bank, expand or reduce the number of active electrodes, and tailor the system to suit specific applications or muscle configurations. This flexibility allows for high levels of personalization while maintaining usability across varying contexts, from communication assistance to motor rehabilitation.

To support this adaptability, user profiles are managed within a centralized server architecture, where they are stored in a versioned database indexed by age and user identity. This design allows for seamless retrieval, updating, and deployment of personalized models and configurations during system use, thereby enabling real-time customization and longitudinally adaptable support.

### 2.9. Server Architecture

The proposed system is deployed on a cloud-based infrastructure, enabling seamless and scalable access for all users. Data acquisition begins with sensor readings collected by an Arduino R4 Wi-Fi module, which transmits these readings, along with timestamps and user identifiers, to a Virtual Private Server (VPS). The machine learning model is hosted on the server and made accessible via FastAPI, allowing efficient, low-latency interactions through an application programming interface (API).

Sensor data is collected via an Arduino R4 Wi-Fi module, which transmits the readings along with a timestamp and user ID to a Virtual Private Server (VPS) using UDP (User Datagram Protocol). Sensor data is transmitted using the User Datagram Protocol (UDP), a lightweight communication protocol suited for time-sensitive data transmission. In UDP, information is sent in the form of discrete packets (datagrams) without the need to establish a persistent connection. This results in faster data transmission, though occasional packet loss can occur. In the context of our application, such losses are acceptable, as rapid data throughput is prioritized over guaranteed delivery. The Arduino transmits data at a frequency of 50 Hz, which is received in real time by a UDP server deployed on the cloud for real-time processing and further transmission.

Upon receipt, the UDP server asynchronously writes incoming data packets to a structured database and simultaneously publishes the data to a NATS (Neural Autonomic Transport System) Publish/Subscribe (Pub/Sub) messaging queue. The Pub/Sub architecture allows multiple services to communicate asynchronously: a publisher sends data to a named topic (or subject), and any number of subscribers can retrieve and process that data independently. This architecture promotes decoupling between system components and supports scalability. In our implementation, the UDP server functions as the publisher, and the CNN-based gesture classification module, exposed via FastAPI, acts as the subscriber.

Once sensor data is received by the FastAPI endpoint, it is passed through the CNN model for real-time gesture inference. The resulting classification outputs are stored in a PostgreSQL database, alongside the original sensor data and relevant metadata for future retrieval and analysis. The system’s database schema will be detailed in a subsequent section.

To provide user feedback and system transparency, a graphical user interface (GUI) has been implemented using Python’s Streamlit package version 1.36 This UI offers real-time visualizations of both the incoming sEMG signals and the corresponding gesture inferences, allowing users and researchers to monitor system performance dynamically.

**Figure 9:**
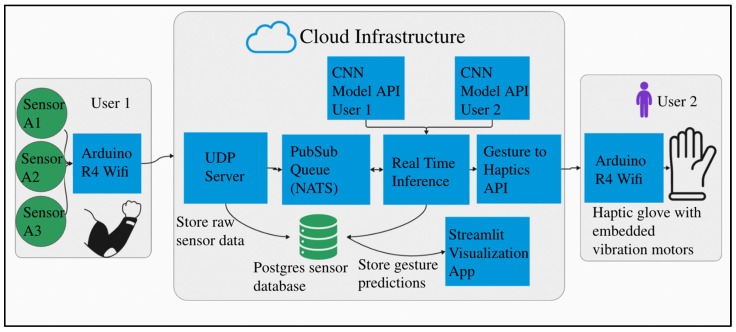
System architecture for multiple users on a centralized server.

Figure 9 illustrates the complete system architecture using three example users. Each user’s data is independently transmitted to the UDP server for processing and analysis. The system accommodates individual variability, including differences in the number of muscle sensors, unique gesture sets, and variations in electrode placement. This adaptability is essential for applications in prosthetics, medical rehabilitation, and personalized communication, where precise muscle targeting may be difficult for users without anatomical training. While the system tolerates minor deviations in electrode location, we recommend adhering to the electrode placement guidelines outlined in earlier sections to ensure optimal accuracy. These recommendations are based on targeting the most relevant forearm muscles responsible for fine finger control.

Our system runs on a commodity Virtual Private Server (2 vCores CPU, 4 GB RAM, 120 GB NVMe disk) with the entire stack dockerized for deployment on any commodity server or cloud platform. The architecture uses FastAPI with NATS pub/sub messaging and PostgreSQL; custom UDP for low-latency streaming; and end-to-end latency <100 ms (5 ms inference + network overhead), measured using timestamp differentials between client request and server response.

### 2.10. Model Personalization

To support longitudinal adaptability, the system allows for the creation of personalized user profiles associated with distinct life stages. Upon initialization of a new stage, a new entry is created in the users table. Each profile is capable of defining a unique set of gestures (user_gestures table) and specifying which sensors are involved in detecting each gesture, offering fine-grained control over signal acquisition.

When the user initiates a training session, the system collects a variety of data streams including gesture labels, training metadata (user_gesture_trainingmetadata), multi-channel sensor readings (user_sensor), and synchronized video frames (user_video). The captured video serves as a critical reference for validating the accuracy of the labeled sensor data, effectively providing a ground truth for the gestures performed.

The labeled dataset is subsequently used to train a personalized convolutional neural network (CNN) in the background. The training process is tracked in real time and recorded in the job_training_metadata table, which stores log messages, training/validation loss metrics, and timestamps for the job’s start and completion.

Upon training completion, all artifacts required for model inference, including model weights, gesture-to-class mappings, scaling parameters, and sensor configuration details, are saved in the “training_job artifacts_table”. The model and associated scaler objects are serialized using Python’s pickle module. Each serialized model occupies approximately 1 MB, ensuring efficient storage and scalability as the number of user profiles grows.

Once deployed, the trained model becomes available for real-time inference. Incoming sensor data is processed by the model, and the resulting predictions are logged in the api_responses table, along with the user ID and corresponding timestamp. These predictions are also displayed in real time on the Streamlit-based user interface, alongside dynamic visualizations of the live sensor data.

**Figure 10:**
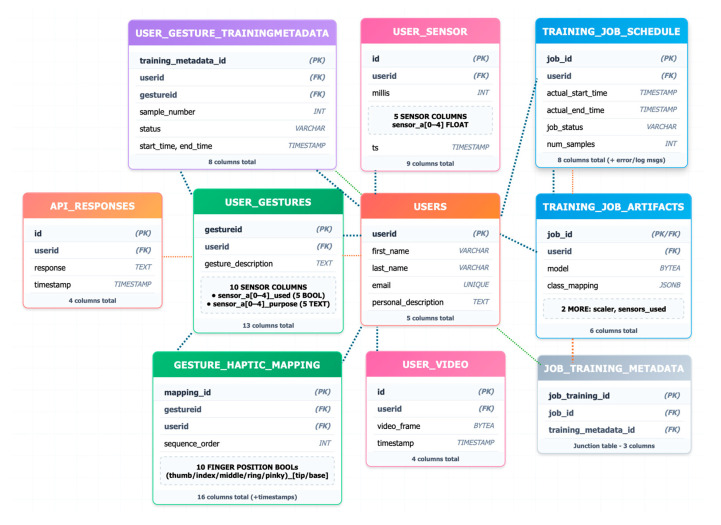
Entity Relation (ER) diagram of database architecture.

Figure 10 illustrates the structure and flow of the underlying database architecture, highlighting the key relationships between user profiles, training data, model artifacts, and real-time system outputs.

### 2.11. Haptic System

An example of a possible haptic system for receiving transmitted communication is illustrated below. The concept mirrors the approach previously demonstrated in [35]. In this system, ten eccentric rotating mass (ERM) coin vibration motors, commonly used in smartphone haptic feedback, are integrated into an arm-mounted sleeve. Each motor is independently addressable and capable of producing distinct vibration sequences. These sequences are designed to convey messages via spatially and temporally patterned tactile stimulation, enabling intuitive, non-visual reception of information by the user.

**Figure 11:**
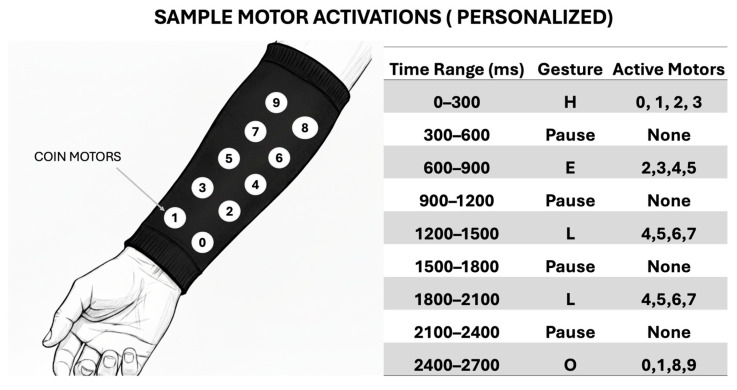
Example haptic encoding for the world “hello”.

Figure 11 illustrates an example encoding of the word “HELLO” using a specific vibration pattern. The haptic patterns are fully customizable, allowing users to define personalized mappings based on different sign languages, dialects, or individual communication preferences.

## 3. Results and Discussion

Since personalized AI algorithms tailored to individual users are a relatively new concept, long-term datasets—spanning multiple years—are currently unavailable. To evaluate the feasibility of this approach, we tested personalized models against a single cross-user model using a publicly available dataset comprising 36 users, as described by Krilova et al. [50].

In this evaluation, model complexity, the number of training samples, and the number of training epochs were held constant to ensure a fair comparison. Results showed that personalized models consistently outperformed the cross-user model, particularly as the number of users increased. For every individual in the dataset, the personalized model achieved higher accuracy than the corresponding cross-user model.

**Figure 12:**
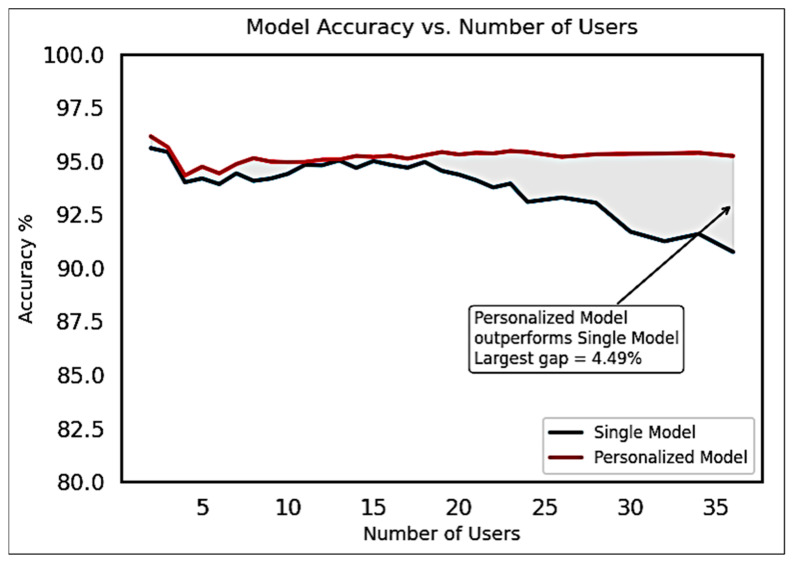
Accuracy improvement observed for multiple Personalized models over Single Cross-User model for various group sizes of users for the Krilova et al. data [50].

To quantify this trend, we trained a cross-user CNN for each subset size from 1 to 36 users and compared its classification accuracy to that of the personalized models. Figure 12 illustrates that the performance gap between personalized and cross-user models increases with the number of users. At the full dataset size of 36 users, the personalized models achieved an accuracy that was 4.49% higher than the single, shared model.

These findings highlight the potential benefits of longitudinally adaptable personalization in sEMG-based gesture recognition systems and support the viability of personalized AI models in real-world, user-specific applications.

To further evaluate the accuracy and practical effectiveness of compact, personalized, single-user models, we conducted a second experiment involving seven human subjects in a live, end-to-end testing scenario. This experiment aimed to assess the real-world performance and adaptability of our system under conditions simulating actual use.

For each subject, we created a new user profile and trained a personalized model using three randomly selected gestures from a predefined gesture bank containing ten distinct hand movements. To collect training data, each subject performed each of the three assigned gestures five times, resulting in a small yet representative dataset per user. Personalized models were trained on this data and immediately deployed for real-time inference. During training, we recorded validation accuracy and validation loss to assess model performance.

To benchmark these personalized models, we also trained a cross-user CNN—with the same architecture—on a combined dataset comprising all users’ gesture data. This model served as a generalized baseline for comparison.

**Figure 13:**
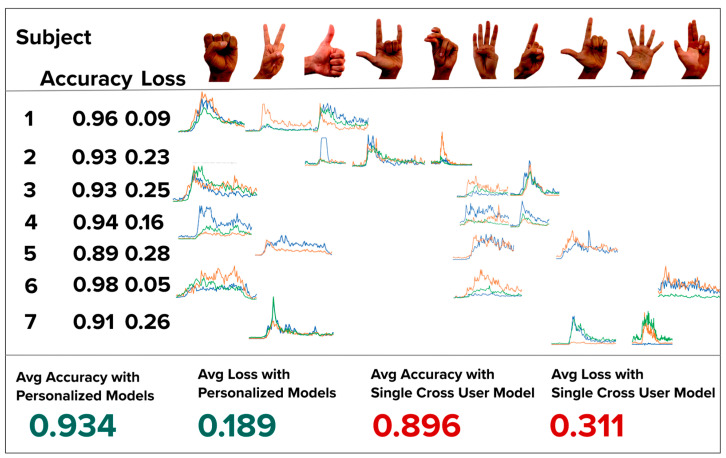
Map of each of the seven subjects and the unique hand gestures randomly chosen. Each hand gesture’s activation data is also shown with 3 channel sEMG data (green, orange, blue). The validation loss and accuracy is presented under each subject. Each personalized model’s validation accuracy is high than the cross-model accuracy.

Figure 13 presents the ten possible hand gestures, the three gestures assigned to each subject, a representative example of the corresponding sEMG activation signals, and the validation accuracy and loss achieved by both the personalized and cross-user models.

As shown, each personalized model outperformed the cross-user model in both validation accuracy and loss, reinforcing the effectiveness of our personalized, low-data approach in an operational end-to-end system.

We hypothesize that the observed decrease in validation accuracy from the personalized models to the cross-user model is primarily due to inter-user variability in both signal amplitude and spatiotemporal characteristics of the sEMG activation data. For instance, subjects from different age groups may exhibit distinct muscle activation strengths, contraction patterns, and timing, all of which contribute to inconsistent input features across users.

These findings support our proposal to use individualized, personalized models to improve gesture inference accuracy, particularly in applications that must accommodate a diverse and heterogeneous user population. Personalized models are inherently better suited to capturing the physiological and behavioral nuances of a single user, thereby reducing classification error.

**Figure 14:**
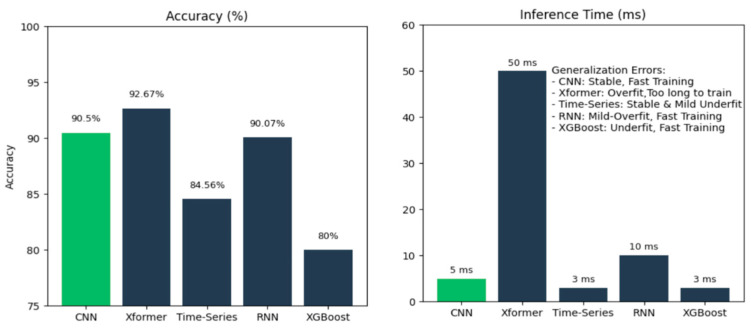
Comparison of accuracy (%) and inference time (ms) for different types of models.

While hybrid LSTM-U-Net architectures could theoretically enhance temporal pattern recognition, our empirical evaluation reveals critical trade-offs, as shown in Figure 14, that favor lightweight CNN approaches for real-time assistive technology. We compared our personalized CNN against several advanced architectures on commodity hardware without GPU acceleration:

These results reveal that for deaf-blind users requiring real-time haptic feedback, the 10× latency difference (5 ms vs. 50 ms) between our CNN and Transformer models is more critical than a 2.17% accuracy gain. Haptic communication demands temporal precision—delays disrupt the natural flow of bidirectional tactile interaction.

Importantly, the lightweight architecture of our personalized CNNs ensures that these models are computationally efficient, with minimal impact on storage or training time. In our experiments, the average model training time was just 107 s, making this approach practical for real-time deployment and long-term adaptation.

### Limitations

While our prototype demonstrates feasibility, we acknowledge critical limitations for daily deaf-blind use:

1. Comfort: Current wet electrodes require 4–6 h replacement and can cause potential skin irritation. Using dry gold-plated electrodes would be a better approach for user comfort.
2. Portability: Current system weighs ~300 g (Arduino R4 + battery + sensors).
3. Battery Life: Currently 2 days of wearable time with 20,000 mAh battery.
4. Setup Complexity: Current 5–10 min electrode positioning is impractical for users with vision loss. Solutions could be using dry electrodes wrapped into a wearable sleeve that has tolerance to placement variations.
5. Sensors: MyoWare 2.0 interface has limitations compared to state-of-the-art systems with custom dry electrodes, lower noise (2.46 μVrms), higher sampling rates (2 kHz), and more channels. Alternative sensing modalities like optical force myography and flexible printed arrays offer potential advantages but remain primarily in research settings. We prioritized commercial availability ($40/sensor) and accessibility for our proof-of-concept. Future iterations will explore these advanced sensor technologies as they mature and become commercially viable while maintaining affordability for the deaf-blind community.
6. Electrode shift—While z-score normalization (Equation (1)) partially mitigates amplitude variations, spatial shifts require robust sensor design and placement guides.

Muscle fatigue was not evaluated as sessions were <1 h, but our low retraining cost (seconds for 1D-CNN) enables periodic recalibration for extended sessions

We also note that testing on the deaf-blind population is necessary to further validate our claims of facilitating deaf-blind communication. In future work, long-term studies with larger numbers of participants would further validate our claims.

## 4. Conclusions

This study introduces a novel, personalized sEMG-based gesture classification system designed to address the unique communication needs of individuals who are deaf-blind, particularly those with progressive conditions such as Usher syndrome. The system’s central innovation lies in its user-specific architecture, which leverages lightweight convolutional neural networks (CNNs) capable of adapting over time to individual physiological changes.

By hosting models on a centralized server infrastructure and employing a lightweight, scalable data pipeline, we enable real-time gesture classification that is both portable and adaptable. Users, such as those with Usher syndrome, can define custom gestures and modify the system to align with changes in muscle anatomy and functional capability over time. The haptic feedback interface, delivered through a wearable sleeve, is also configurable to individual user preferences, supporting further personalization.

Experimental results demonstrate that personalized models significantly outperform cross-user models in both accuracy and adaptability, even when trained on limited data. Our end-to-end system, encompassing data acquisition, preprocessing, model training, inference, and real-time visualization, has been validated using both a public multi-user dataset and a live user study. In addition, relocating the haptic interface from the hands to the arm preserves manual dexterity and increases autonomy, enhancing the overall user experience.

The implications of this work extend beyond the deaf-blind community. The system architecture is readily adaptable for applications in neuroprosthesis, stroke rehabilitation, physical therapy, and other domains where long-term physiological adaptation and personalized response are essential. Future research will focus on improving signal robustness, implementing transfer learning strategies, such as Low-Rank Adaptation (LoRA) for rapid retraining, and enhancing discrimination between intentional gestures and involuntary muscle activations.

By integrating haptics, AI, and electrophysiological sensing, this platform represents a foundational step toward a new generation of inclusive, long-term assistive technologies.

## Data Availability

The data presented in this study are available on request from the corresponding author subject to approval by the UC Berkeley Committee for Protection of Human Subjects ( CPHS), (Protocol 2020-08-13529).
